# Care-seeking pathways, care challenges, and coping experiences of rural women living with rheumatoid arthritis in Odisha, India

**DOI:** 10.1017/S146342361900032X

**Published:** 2019-07-30

**Authors:** Sanghamitra Pati, Krushna Chandra Sahoo, Mousumi Samal, Sunita Jena, Pranab Mahapatra, Deepika Sutar, Bidyut K. Das

**Affiliations:** 1 Regional Medical Research Centre, Indian Council of Medical Research, Bhubaneswar, Odisha, India; 2 Department of Psychiatry, Kalinga Institute of Medical Sciences, Bhubaneswar, Odisha, India; 3 Indian Institute of Public Health, Bhubaneswar, Odisha, India; 4 Clinical Immunology and Rheumatology, Department of Medicine, SCB Medical College and Hospital, Cuttack, Odisha, India.

**Keywords:** care-seeking pathways, India, primary health care, rheumatoid arthritis

## Abstract

**Aim:**

The aim of the study was to explore the care-seeking pathway of rural women living with rheumatoid arthritis (RA) and attending a tertiary health-care facility in Odisha, India.

**Background:**

RA is the third leading chronic health condition and causes severe pain and immense psychosocial stress. The prevalence of RA is three to four times higher in women than in men. Furthermore, in India, women delay care seeking due to the prevailing sociocultural norms. Women report more severe symptoms and greater disability; however, there is a lack of information on their care-seeking pathways.

**Method:**

We conducted 113 in-depth interviews among RA patients those who visited specialists at the outpatients’ Department of Rheumatology, SCB Medical College Hospital, a tertiary care hospital in Cuttack, Odisha, India. The grounded theory approaches were used for data analysis.

**Findings:**

The key findings included physical pain and psychosocial stress in relation to RA, cultural issues in relation to RA, mapping of the health-care providers for RA, the first point of cares and changes in care-seeking pathways, the perceived challenge for seeking health-care, and coping strategies of patients and social supports. This study explored that the RA patients seek care from multiple providers – untrained, trained and specialist without any gatekeeping. However, the primary health centers were the first point of care for maximum patients due to accessibility and affordability. Furthermore, follow-up care is significant to prevent complication among RA patients; the primary health centers are the gateway for keeping RA patients. Hence, the availability of RA trained providers at primary health center including interprofessional care, such as physiotherapy providers, and proper referral system is essential to convalesce care-seeking pathways.

## Introduction

Rheumatoid arthritis (RA) is one of the leading causes of chronic morbidity (Rudan *et al*., 2015). It causes severe pain, swelling, stiffness (Gibofsky, 2014), and extra-articular comorbidities and results in productivity loss among patients (Smith *et al*., 2014). The prevalence of RA is three to four times higher in women than in men (Handa *et al*., 2016). A previous study in Odisha revealed that RA is the third leading cause of chronic disorder. Its prevalence is higher among women (18%) than men (13%; Pati *et al*., 2017). Furthermore, it is more common among rural women (AHS, 2012).

Community members’ misbelieve on RA and their own interpretations of symptoms are key roadblocks for health-seeking behaviors (Stack *et al*., 2012; Smith *et al*., 2014; Rudan *et al*., 2015). The meta-ethnography studies revealed that patients’ noncompliance behavior often delayed diagnosis (Stack *et al*., 2012). The use of complementary and alternative medicine (CAM) largely from the multiple conventional providers is very high among RA patients (Yang *et al*., 2017). In India, most of the RA patients seek care from untrained or traditional providers to get instant relief (Deshmukh *et al*., 2014). Moreover, Indian rural women often delay in seeking care due to the prevailing sociocultural norms.

The care-seeking pathway (CSP) studies on some chronic disorders, such as patient navigation pathway in cancer (Pati *et al*., 2013), pathways to care among psychiatric outpatients (Jeyagurunathan *et al*., 2018), and parental CSP in autistic spectrum disorders (Mahapatra *et al*., 2019), are the evidence for the significance of CSP studies. However, there is a paucity of information on CSPs, care challenges, and coping experiences among RA. Therefore, this study aims to explore the CSP of rural women living with RA.

## Methods

This study was conducted in Odisha, India, a state with a total population of 41 million; of which about 83% are living in rural areas. It was carried out at the outpatients’ Department of Rheumatology, SCB Medical College Hospital, a tertiary care hospital in Cuttack, Odisha, India. We conducted 113 in-depth interviews (IDIs) among rural women patients who had visited RA specialists for treatment. We collected sociodemographic information and CSPs, that is, types of health-care providers, and average times of visit before reaching rheumatologist from the onset of the disease for mapping. The average age of the participants was 38 years with a range of 30–49 years; 13 participants had no formal education, 54 had primary education, 33 had high school education, and 13 were graduates; 85 were homemakers, 21 labors and 7 had private jobs. We carried out five to six IDIs in a week by contacting 8–10 participants; some of them did not agree because of their time constraint. The last author facilitated the recruitment of the participants. The IDI guides adapted throughout the data collection process was based on emergent findings. The IDI guide is presented in Table 1.

**Table 1.** In-depth interviews’ (IDIs) guide

Describe the prediagnosis history of your disease (RA)? Probe: First experience, perception, and beliefs about the disease and treatment, consultation, and delay in seeking care Could you share your experience on diagnosis? Probe: Appointment, diagnosis process, expenditure, referral, and problems faced during diagnosis How did you maintain your disease condition as well as treatment process? Probe: Frequency of visit to doctor, barriers, enablers, coping How is your social environment related to this disease condition? Probe: Workplace challenge, neighbor, challenges in daily life, family support

All the IDIs were digitally recorded, hand transcribed in Odia, and reviewed for accuracy by the trained interviewers. Transcripts were then translated into English by interviewers fluent in English and Odia. As a quality control measure, randomly selected English-language transcripts were reviewed simultaneously with the Odia recording and documents corrected appropriately. In this study, we used the principles of grounded theory approaches (Charmaz, 2014). Participant selection, data collection, and analysis were done by an iterative process. Subsequent data collection and analysis were responsive to emergent categories and concepts. The findings were identified inductively through data collection and analysis. During data collection, the study team read transcripts and prepared initial notes and summaries of the interview. Line-by-line open-coding was conducted using MaxQDA (Berlin, Germany). The authors K.C.S., M.S., S.P., and S.J. independently analyzed the text and finally all the codes and categories were triangulated by K.C.S. and S.P., and M.S. prepared the brief summary of each category. The codes were identified and the codes were categorized based on their similar characteristics. After the IDIs, all authors debriefed the transcripts and reviewed the CSPs and decided the potential participants for the next round of IDIs. Participants were selected from various age categories and educational background to bring variations in the information. Interviews were conducted in Odia by the trained female interviewers (D.S. and M.S.) who are fluent in their local language. The interviews ranged from 15 to 45 minutes (mean duration 25 minutes). Data saturation was achieved after 113 IDIs as most of the IDIs were of short durations.

This study was approved by an ethical review committee of XXXXX. The permission was obtained from the concerned local authority. All the participants participated voluntarily; payment was neither offered nor given. Before the interview, information was given about the purpose of the study to the participants and they were told that they could withdraw from the study at any time. All the interviews were carried out in complete privacy at the place chosen by the participants and their names were kept confidential. The written consent was obtained from literate participants and thumb impressions from the illiterate participants.

## Findings

### Physical pain and psychosocial stress in relation to RA

Physical pain was the major concern for RA patients. Most of them suffered unbearable pain from leg to waist. The severity of pain was intolerable during evening and winter. They struggled in doing their daily activities such as wearing a dress, washing clothes, and doing household works. Some of them were unable to lift heavy things and carry their kids. Most of them felt difficulties in mobilizing limb, getting up, sitting down, standing properly and getting up from the toilet.

How much difficulties it may be, I can’t avoid urination and defecation. I urinate outside the drain by standing, it was shameful, but what can I do; I feel more difficult during defecation. I can’t wear saree and comb hair, even I am unable to breastfeed my baby.

### Cultural issues in relation to RA

Most of the patients viewed that along with severe pain, the community members’ negative remarks about the disease increased their fear, which resulted in mental stress. The community members remarked that RA is incurable and leads to paralysis and that allopathic treatment is ineffective.

Neighbour told that my disease is not curable, I will get paralyzed and I have to take medicine for a lifetime. They said eat herbs don’t take allopathic medicine, it won’t help; I am staying alone here, that increase my stress.

According to some community members’ perception, RA occurred because of wrong deeds in the previous birth of the person, which in turn causes social stigma. Some of the patients alleged that suffering from RA and living alone without any support from the family is another factor for mental stress.

I am a widow; no one is here to look after me (sobbing). I have two sons; they are doing jobs and staying away from me.

Most of the participants experienced that being a female and suffering RA is more painful because of our societal structure females have more responsibility in household work and child caring. According to some patients in a joint family, it is easy to get social support; however, they have to adjust to other member’s needs.

They (husband and children) sleep under air cooler, I switch it off, and they became irritated. I feel cold and they feel hot; we have only one bedroom.

### Mapping of the health-care providers for RA

The patients seek care from three types of service providers: untrained, trained, and specialist. The untrained providers were divided into two categories – the first category included home-based treatment, indigenous care, and traditional healers; and the second category included informal health-care providers and over-the-counter drugs from the pharmacy shop.

There were three types of trained providers. The first group of trained providers belonged to Ayurveda, Yoga, Unani, Siddha, and Homoeopathy (AYUSH) health-care systems (Indian alternative health-care system); the second group were trained allopathic providers (general practitioners) at primary health centers and private clinics; and the third group included providers at community health centers and subdivisional hospitals.

Specialists or rheumatologists were available at district, capital hospital, medical college, and corporate hospitals. In every district, there is one district hospital; the average distance of district hospital from the village was 40 km, with a range of 10–80 km. Corporate hospitals were located only in the city.

### The first point of care and changes in CSPs

**Figure 1.**
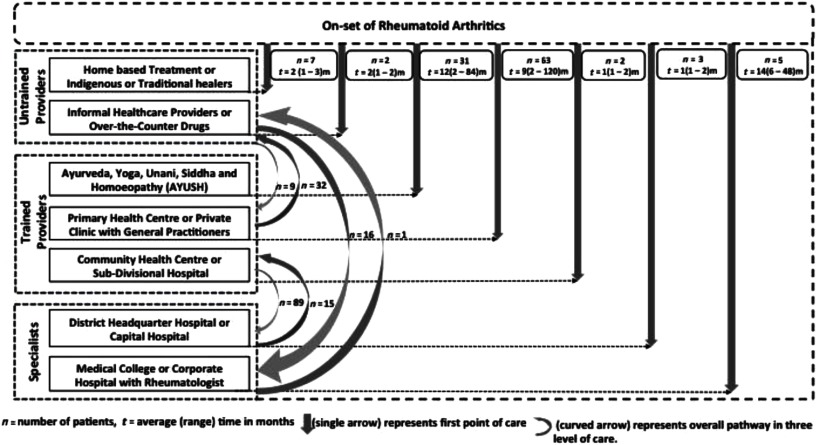
Care-seeking pathways of female rheumatoid arthritis patients before reaching rheumatologist.

A conceptual framework on CSPs was developed using IDIs’ information obtained from 113 female RA patients from a specialist. Figure 1 describes the CSPs of female RA patients before reaching a rheumatologist in a tertiary health-care facility.

The single arrow in Figure 1 showed the patients’ care-seeking practice from the onset of RA to the first point of care. The number of patients and total duration of treatment at the particular provider/facility before changing to another provider/facility were provided in a box attached to the respective arrow. The study explored that 8 patients seek the first point of care from specialists, 96 from trained providers, and 9 from untrained providers. The curved arrow in Figure 1 represents the change in CSP between three levels of health-care providers: untrained, trained, and specialist at any point in time. Among the patients visiting a specialist, 8 directly visited them, 89 visited after consulting the trained providers, and 16 visited after consulting untrained providers.

### The perceived challenge for seeking health care

All the patients had accepted the treatment of specialists, only 8 patients had a barrier to accepting treatment from trained providers, and 81 patients did not accept the treatment provided by untrained providers. According to all the patients, no one had affordability issues to seek care from trained providers at public health facilities. However, 32 patients viewed that the treatment from untrained providers was expensive particularly from traditional healers as they prescribed high-cost traditional medicines.

The medicines provided by traditional healer were bitter than sugar; after taking that medicines, my tongue turned black. I spent 4000 Rupees per month for medicine, nothing got better, and rather I became weak, so I stopped taking the medicines. Even my neighbors suggested me for grey horn-bill bird (Kochilakhai) which cost around seven thousand rupees for one bird and also 64 types of spices needed for its preparation.

Above two third of the participants viewed that poor financial conditions were key barriers to consulting specialists, as they need to bear travel and accommodation cost at the lodge during a visit to specialists. Furthermore, the medicines prescribed by specialists were expensive.

I have a beetle shop; my monthly income is six thousand rupees; I spent so much for this disease. I borrowed money from friends. The doctor (specialist) prescribed expensive medicine. How long will I depend on others! That worried me a lot.

According to all patients, untrained providers were easily accessible. Many patients opined that the long distance to visit a specialist and extensive waiting time because of patient load were key challenges to seek care from specialists (Table 2).

At specialist, the crowd is more. I came here thrice, but unable to meet the doctor. Once I had 145 serial numbers, I waited a long time then left. Today, my serial number is 220; I have made up my mind that I will go only after consulting with him. The lodge and food cost is also a burden for me.

**Table 2.** Perceived barriers to health-care services from three types of providers among female rheumatoid arthritis patients

	Acceptability, *n*	Affordability, *n*	Accessibility, *n*
Specialists	0	57	105
Trained providers	8	0	32
Untrained providers	81	32	0

### Coping strategies of patients and social supports

Most of the patients viewed that they were coping with RA by doing meditation (Yoga and Pranayama) and light exercise and avoiding oily and spicy food. They were using traditional treatments, oil massage, and rubbing ice on pain areas to reduce the severe pain. Some of the patients coping with RA avoid the use of fans and air condition room but use a blanket at night, bath with hot water, and use the western toilet (commode). Half of the participants said that they use steroids and painkillers to get relief from pain.

Every day, I used to have a painkiller, without painkiller I am unable to do any work, everything is done after taking painkillers, I buy painkiller from medicine shop.

Many patients in our study revealed that they borrowed money from relatives and friends for the treatment of the RA. According to some patients’ experience, their stress due to RA could be reduced, if they had extra money after maintaining their family or the health insurance for the treatment. Some patients reported that that they took the help of mobile phones, computers, and other social media to get information on relief from the pain due to RA. According to most of the patients, the family members’ understanding and empathy toward the disease was a significant factor. All the patients expected support from husband and other family members in their routine work, household activities, and treatment.

My children used to massage me with oil, bring painkiller ointments. My daughter-in-law does all the work. I bath with hot water and use a blanket at night. As I am living in a joint family, everyone understands the disease, so I don’t face more problems.

Some patients mentioned the importance of doctors’ behavior and guidance, referral system to specialists, and availability of more numbers of specialists for better care.

I went to a community health center, the doctor told it’s not good to have these symptoms; after childbirth, I may face more problems. I was asked to do a test and consult a specialist at a nearby town. The doctor is very good and his behavior is good.

## Discussion

To the best of our knowledge, this is the first study exploring the CSPs of RA patients. The Sustainable Development Goal 3 (SDG3) relates to ‘Ensure healthy lives and promote well-being for all at all ages’. The SDG3 is translated into 13 targets, and the universal health coverage (UHC) is one of the targets. The primary health care is significant and vital for its cross-cutting role to achieve UHC (Pettigrew *et al*., 2015). The primary health centers were the first point of care for maximum patients, as it is easily accessible, affordable, and acceptable. However, most of the patients in this study preferred multiple pathways to seek care because of severe pain and psychosocial stress.

The physical pain and psychosocial stress were the major concerns among RA patients (Gibofsky, 2014; Smith *et al*., 2014), which is similar to our findings. Most sufferers develop the RA between 35 and 45 years of age and many of them have experienced functional loss and working disability (Scott *et al*., 2010); and at the same time, they seek care from multiple providers including CAM providers (Yang *et al*., 2017).

In our study, the patients had CSPs to multiple providers such as untrained, trained, and specialist without any gatekeeping. One -third of the patients sought care from untrained providers. The review shows that there was a high prevalence of multiple types of CAM use among RA patients worldwide, which was not limited to specific socioeconomic groups or geographic locations (Geisler and Cheung, 2015; Yang *et al*., 2017). According to Barnes *et al*., above half of the Americans use CAM to manage various medical conditions, and one third of them mention pain as the primary reason (Barnes *et al*., 2008). Conversely, two thirds of them never mention these products to their health-care providers (Yang *et al*., 2017). As a consequence, an invisible ‘mainstream’ of alternative care exists, and little is known about its safety, efficacy, and mechanism of action. Our study explored people centeredness in health systems in RA care, and half of the patients used an analgesic without prescription to get relief from the pain.

The people centeredness in health systems is an emerging public health challenge (Sheikh *et al*., 2017). In our study, in relation to care from specialists, most of them visited specialists after seeking care from multiple providers. Meanwhile, all the patients accepted the treatment of specialists. One third of the patients viewed that the treatment from untrained providers was also expensive, though they were easily accessible, which is similar to other findings (Yang *et al*., 2017). Most of the patients in our study had the challenge to access specialists; being females they often needed a male person to accompany them to visit a specialist.

Gender has remained as a predictor of RA outcomes over the decades. Recent reports suggest that females are less likely than males to achieve remission (Sokka *et al*., 2009). Women were more frequently affected compared to men and therefore have a significant impact on their physical and emotional well-being and social functioning (Sokka *et al*., 2009). Women report more severe symptoms, greater disability, and higher work disability rates compared to men (Lesuis *et al*., 2012). Moreover, in a country like India, women often face a challenge for seeking appropriate care because of patriarchy social system and *pluralistic* health-care system.

The *pluralistic* health-care system is ignored in countries where much effort has been put into building a single, uniform health service delivery system. However, there is compelling evidence that for many health problems, households do not automatically avail themselves of the public health services, often preferring self-medication, traditional, and private providers (Meessen *et al*., 2011). India has a pluralistic medical culture with a well-documented history and practice of alternative medicine – AYUSH and Naturopathy (Rudra *et al*., 2017). Meanwhile, a lack of understanding of the pluralistic medical system has impeded programs to improve community health status in India. Assumptions and misconceptions about the pluralistic Indian medical system are examined and their implications for health service are assessed. It was found that preference for practitioners from their own cultural background is less important to patients than their availability, accessibility, and quality of care provided by different systems. When they lack easy and adequate access to modern care, people rely on traditional healers in primary care (Meessen *et al*., 2011). Hence, this study emphasizes the need for RA management at the primary health centers and proper referral system to gatekeep the patients who are seeking care from multiple providers including untrained providers and traditional healers.

To improve the trustworthiness of our study, we used data triangulation during the analysis. Data were collected from different categories of RA patients. During the coding procedure, both Odia and English versions of the transcripts, and in some complex cases tape recorded data, were used simultaneously to avoid misinterpretation of the full meaning. The authors of this study were from different educational backgrounds. Hence, each author brought a unique perspective to the study, enhancing its conformability. To avoid the culturally sensitive issue, we followed member check methods (revisiting few participants after finishing the analysis), suggesting that that their perceptions were interpreted correctly. The limitation of this study was that though the focus group discussion (FGD) is considered better for data collection. As our study setting was a tertiary care hospital, it was difficult to organize FGD. Another limitation of the study was the initial IDIs were of short duration; hence, we conducted more IDIs to reach data saturation. Although we conducted the study in Odisha, the findings might be useful in other similar settings.

## Conclusion

This study explored that the RA patients seek care from multiple providers – untrained, trained and specialist – without any gatekeeping. However, due to its accessibility and affordability, the primary health centers were considered the first point of care for most patients. Furthermore, follow-up care is significant to prevent complication among RA patients. The primary health centers are the gateway for keeping RA patients. Hence, the availability of RA trained providers at primary health center including interprofessional care, such as physiotherapy providers, and proper referral system are essential to convalesce CSPs. This study also suggests that RA should be included in the noncommunicable disease policy for better health and wellness among RA patients.
